# A knowledge translation intervention to improve tuberculosis care and outcomes in Malawi: a pragmatic cluster randomized controlled trial

**DOI:** 10.1186/s13012-015-0228-y

**Published:** 2015-03-28

**Authors:** Lisa M Puchalski Ritchie, Michael J Schull, Alexandra LC Martiniuk, Jan Barnsley, Tamara Arenovich, Monique van Lettow, Adrienne K Chan, Edward J Mills, Austine Makwakwa, Merrick Zwarenstein

**Affiliations:** Department of Medicine, University of Toronto, Toronto, Ontario Canada; Department of Emergency Medicine, University Health Network, Toronto General Hospital, RFE-GS-480, 200 Elizabeth St., Toronto, Ontario M5G 2C4 Canada; Institute of Health Policy, Management and Evaluation, University of Toronto, Toronto, Ontario Canada; Dignitas International, Toronto, Canada; Sunnybrook Health Sciences Center, Toronto, ON Canada; George Institute for Global Health, Sydney, Australia; The University of Sydney, Sydney, Australia; Dalla Lana School of Public Health, University of Toronto, Toronto, Canada; Dignitas International, Zomba, Malawi; Faculty of Health Sciences, University of Ottawa, Ottawa, Canada; National TB Control Program, Ministry of Health, Lilongwe, Malawi; Knowledge translation unit, Lung Institute, University of Cape Town, Cape Town, South Africa; Stellenbosch University Faculty of Health Sciences, Tygerberg, South Africa; Depart of Family Medicine, Western University, London, Ontario Canada

**Keywords:** Lay health workers, Community health workers, Educational outreach, Reminders, TB, Cluster randomized trial

## Abstract

**Background:**

Lay health workers (LHWs) play a pivotal role in addressing the high TB burden in Malawi. LHWs report lack of training to be a key barrier to their role as TB care providers. Given the cost of traditional off-site training, an alternative approach is needed. Our objective was to evaluate the effectiveness of a KT intervention tailored to LHWs needs.

**Methods:**

The study design is a pragmatic cluster randomized trial. The study was embedded within a larger trial, PALMPLUS, and compared three arms which included 28 health centers in Zomba district, Malawi. The control arm included 14 health centers randomized as controls in the larger trial and maintained as control sites. Seven of 14 PALMPLUS intervention sites were randomized to the LHW intervention (PALM/LHW intervention arm), and the remaining 7 PALMPLUS sites maintained as a PALM only arm. PALMPLUS intervention sites received an educational outreach program targeting mid-level health workers. LHW intervention sites received both the PALMPLUS intervention and the LHW intervention employing on-site peer-led educational outreach and a point-of-care tool tailored to LHWs identified needs. Control sites received no intervention. The main outcome measure is the proportion of treatment successes.

**Results:**

Among the 28 sites, there were 178 incident TB cases with 46/80 (0.58) successes in the control group, 44/68 (0.65) successes in the PALMPLUS group, and 21/30 (0.70) successes in the PALM/LHW intervention group. There was no significant effect of the intervention on treatment success in the univariate analysis adjusted for cluster randomization (*p* = 0.578) or multivariate analysis controlling for covariates with significant model effects (*p* = 0.760). The overall test of the intervention-arm by TB-type interaction approached but did not achieve significance (*p* = 0.056), with the interaction significant only in the control arm [RR of treatment success for pulmonary TB relative to non-pulmonary TB, 1.18, 95% CI 1.05–1.31].

**Conclusions:**

We found no significant treatment effect of our intervention. Given the identified trend for effectiveness and urgent need for low-cost approaches to LHW training, further evaluation of tailored KT strategies as a means of LHW training in Malawi and other LMICs is warranted.

**Trial registration:**

ClinicalTrials.gov NCT01356095.

## Background

Globally, 1.7 million lives were lost to TB in 2009 [1], with mortality rates up to four times greater in individuals co-infected with HIV [2]. Among the 20,342 new TB case notifications in Malawi in 2010, approximately two thirds were co-infected with HIV [3]. Despite ongoing efforts, poor adherence remains an important contributor to the high TB burden in Malawi, with default rates for TB and antiretroviral treatment as high as 16% [4], and multidrug-resistant TB accounting for 2.3% of new and 7.5% of recurrent TB cases [5].

An estimated 4.2 million skilled health care workers, 1.5 million in Africa alone, are needed to address the chronic global shortage of skilled health workers [6]. Shifting of less complex health care tasks to lower cadres of health workers, coined task-shifting, is an increasingly employed strategy to addressing this shortage [7], particularly in low- and middle-income countries (LMICs). Lay health workers (LHWs), who are ‘members of the community who receive some training to promote health and to carry out some health services but are not healthcare professionals’ [8], are now widely involved in healthcare in Africa. Although shown to improve access to preventative and basic health services, and positively impact some health outcomes [8,9], insufficient training and supervision are recognized barriers to the effectiveness of LHW programs [9]. Despite their widespread involvement in healthcare, evidence on how LHW training and supervision needs may best be met is lacking [7].

Although approaches to training in lay health worker programs vary considerably [9], ongoing training frequently involves removing LHWs from the workplace to receive training off-site [10]. This approach is problematic because it is expensive in terms of both financial and opportunity costs [10] associated with the disruption of care provision. Given the resource implications and lack of evidence for effectiveness of this approach to ongoing training, research to design and evaluate alternative approaches to training are needed [7].

Malawi has among the lowest healthcare worker to population ratios with 2.03 physicians and 36.8 nurses per 100,000 population in 2009 [11]. In response to this crisis, a variety of basic health care tasks have been shifted to a formal cadre of paid lay health workers termed health surveillance assistants including provision of outpatient TB care and adherence support. At the time of this study, pre-service training for general LHWs consisted of 10 weeks of in-class training, with approximately 1 day devoted to TB control, transmission, and treatment. A subgroup of LHWs, termed TB focus LHWs, receive 2 weeks of additional TB specific training and are responsible for the provision of TB care at the health center level. TB focus LHWs recruit and train general LHWs to assist with TB care. The content and duration of training is at the discretion of the individual TB focus LHW and ranges from a 1–2 hour briefing to several days working alongside the TB focus LHW while they provide TB care.

LHWs have a pivotal role in addressing the high TB burden in Malawi, with over 20,000 new TB notifications in 2010 [3]. In a recent study we conducted with LHWs in Zomba district, we found a lack of initial and ongoing training to be the key barrier to LHWs in their role as TB care providers and adherence supporters [12]. Given the cost implications of the typical off-site training approach with over 10,000 LHWs currently working in Malawi [11], an alternative is essential to ensure that training needs are met. Based on the relative low cost and proven success of knowledge translation (KT) strategies, as a training and clinical support tool for mid-level health workers in South Africa [13-15] and in the KT literature in general [16,17], we aimed to determine the effectiveness of a KT intervention tailored to address the TB training needs of LHWs in Malawi using a cluster randomized trial design.

## Methods

### Study design

We conducted a pragmatic cluster randomized trial to evaluate the effectiveness of a knowledge translation intervention tailored to the needs of LHWs to improve TB treatment adherence. TB care is provided within a given health center by LHWs on a rotating basis, with LHWs assigned to the health center TB office in weekly blocks at many health centers. As a result, patients typically receive care from a number of LHWs over their course of treatment. For this reason, a cluster design, with health centers as the unit of allocation and TB patients as the unit of analysis, was chosen in order to prevent contamination and to optimize the benefits of the intervention at the patient level.

### Setting and participants

Our cluster randomized trial was embedded within a larger cluster trial, practical approach to lung health plus HIV/AIDS in Malawi (PALM PLUS) [18]. The PALM PLUS intervention employed an integrated guideline adapted to Malawian protocols for adults with respiratory conditions, HIV/AIDS, tuberculosis, and other primary care conditions and a training program for [19] mid-level health workers (nurses and clinical officers) in Zomba district health centers. Of the 30 health centers included in the larger trial, 28 routinely provided care to TB patients and were eligible for inclusion in the present study. In order to prevent contamination of the larger study by introducing an intervention at control sites, only the 14 PALM PLUS intervention sites were eligible for allocation to the LHW intervention. All LHWs routinely involved in providing care to TB patients were eligible and invited to participate in the intervention, with refusal to participate the only exclusion criteria.

### Randomization

Health centers were randomized into three arms: control arm, PALM PLUS only arm, and PALM PLUS with LHW intervention arm. The 14 PALM PLUS intervention sites were randomly allocated centrally by a research assistant using a computer-generated random numbers list, stratified based on whether the health centers were antiretroviral therapy (ART) initiation sites at the time of randomization. This stratification was chosen as many LHWs involved in TB care, also provide services to HIV patients, with the potential for LHWs at ART initiation sites to have received additional clinical skills training. Given the nature of the intervention, blinding was not possible. The 14 remaining district health centers involved in provision of care to TB patients remained control sites as previously allocated in the PALM PLUS trial.

The study was approved by both the Malawi National Health Sciences Research Committee and the University of Toronto Research Ethics Board. Individual consent was not required from health center staff, as the intervention was developed in collaboration with and approved by the national TB program, and while participation in training is a routine expectation of health center staff, participants were invited. As participation in work-related training is often a requirement, it was made clear to LHWs that participation in this project was optional and that non-participation would not affect their job or performance appraisal. All outcome data were taken from routinely collected Ministry of Health records, with consent from individual patients not required.

### Intervention

The intervention was designed to address evidence on risk factors for non-adherence from the literature and to address a recognized gap in TB care provided by LHWs in Malawi identified through stakeholder consultations, field observation, and a qualitative study conducted with LHWs providing TB care in Zomba district [12]. The LHW intervention employed two KT strategies proven effective in changing provider behavior, specifically educational outreach and reminders, with the goal of increasing the uptake of evidence on barriers to adherence and targeting these through changes in provider behavior designed to address known barriers and improve outcomes.

The educational outreach component employed peer trainer-led on-site training, using a combination of didactic and interactive techniques including case-based discussions and role playing to efficiently provide TB-specific knowledge and adherence counseling skills and to allow for practice and exchange of ideas between LHWs. Topics were initially chosen based on the adherence literature and expanded to address the training needs identified by Zomba district LHWs in a prior study conducted by our group [12]. Topics included: TB transmission, natural history, treatment, and consequences of poor adherence; the interaction of TB and HIV; and common barriers to adherence and appropriate methods for preventing and addressing non-adherence (see detailed description in Table 1). The second component was a point-of-care clinical support tool (reminder) designed as a laminated chart that can be folded and carried during field visits or stand on the desk to be referenced during patient interactions. One side of the tool provides a visual reminder designed to trigger an adherence discussion during patient interactions and provides clinical support for management of side effects and a constructive approach to addressing issues with adherence. The opposite side uses simple pictorials to illustrate key messages used in patient education and adherence counseling. The tool was pilot tested with LHWs providing TB care at the district hospital.

**Table 1:** **Description of the intervention**

**Details of intervention**	**LHW intervention group**
Rationale/goals	The intervention was designed to target a recognized gap in TB care provided by LHWs by targeting two common barriers to adherence, patient disease understanding, and patient-provider relationship through improved LHW TB knowledge and skills in patient education and adherence counseling.
Materials	The educational outreach component utilized a combination of didactic and interactive techniques including case-based discussions and role playing to efficiently convey TB-specific knowledge and adherence counseling skills and to allow for practice with the point-of-care tool and exchange of ideas between LHWs. Topics included: TB transmission, natural history, treatment, and consequences of poor adherence; the interaction of TB and HIV; and common barriers to adherence and approaches to preventing and addressing non-adherence while maintaining a positive patient-provider relationship.
	The point-of-care tool is designed as a laminated chart that can be folded and carried during field visits or stand on the desk to be referenced during patient interactions. One side of the tool provides a visual reminder designed to trigger an adherence discussion during patient encounters and provides clinical support for management of side effects and a constructive approach to addressing issues with adherence. The opposite side uses simple pictorials to illustrate key messages used in patient education and adherence counseling. The tool was pilot tested with LHWs providing TB care at the district hospital.
	Both the training manual and point-of-care tool are available at Development and Evaluation of a Tailored Knowledge Translation Intervention to Improve Lay Health Workers Ability to Effectively Support TB Treatment Adherence in Malawi. http://hdl.handle.net/1807/35187
Procedures	Peer-led educational outreach sessions occurred on-site at participants’ base health center during regular work hours. Peer trainers provided a minimum of six sessions, each 60–90 minutes in duration, over a 3-month period.
Intervention provider	TB focus LHWs, general LHWs with 2 weeks additional TB training who are responsible for TB care at the health center level, trained as peer trainers.
Method of delivery	Face to face
Location/context	Sessions took place at the LHWs base health center during regular work hours.
Intensity	6 sessions, each lasting 60–90 min, over a 3-month period.
Tailoring	Additional sessions as makeups for staff that missed sessions, for extra practice as requested of the peer-trainer by the local TB team, or to discuss difficult cases/share experiences within the LHW TB team, were left to the discretion of the peer trainers.
	All sites reported meeting at least quarterly to discuss cases, and many reported making up sessions for staff that missed sessions due to illness or leave.
Modifications	Training period extended from 2 to 3 months to accommodate staff absences due to annual leave/illness.
Fidelity	As this was a pragmatic trial, fidelity was not formally assessed due to concerns such assessment could act as boosters to the intervention, which would not occur under real world conditions if scaled up.
	Informal reports from peer trainers and LHW participants during quarterly meetings, field visits, and interviews in a companion qualitative study indicated a small number of participants (estimated at 4–5) did not complete the full curriculum.

Peer LHW trainers were selected in consultation with the district environmental health office. TB focus LHWs are general LHWs who have received 2 weeks additional TB specific training and are responsible for provision of TB care at their health centers. General LHWs assigned to assist in providing TB care are in turn trained by the TB focus LHW with the content and duration of this training left to the discretion of the TB focus LHWs.

Peer trainers participated in a 3-day training course in March covering both the content of the training itself as well as techniques for peer training. Trail registration was delayed approximately 6 weeks, as contact was lost with one of the trainers for almost 2 months during which time we believed an alternate trainer would have to be recruited and trained and as a result delay the trial start date. However, contact was regained in mid-May and the trial registered at this time, and data included from the original planned start date.

Peer trainers were asked to provide a minimum of six sessions of 60–90 minutes in length over a 2-month period during regular work hours to all the general LHWs routinely involved in provision of care to TB patients at their health centers. Timing of sessions was left to the discretion of the trainer and their team. The training period was extended to 3 months to allow for a second block of training at sites with large numbers of LHWs. All trainers reported providing the minimum number of sessions as outlined in the training manual, with two trainers reporting providing additional sessions to train additional providers or as makeup sessions for LHWs who missed sessions. Training materials were provided, but no incentives were provided to trainers since training of general LHWs assigned to assist with TB care is part of their usual job description, and payment of incentives would limit sustainability. Peer trainers were brought together quarterly to provide an opportunity to address any questions or concerns with the research team and to allow for exchange of experiences with the initial training and ongoing use of the point-of-care tool.

Lay health workers in the control arms received the usual training at the discretion of their TB focus LHWs. The PALM PLUS intervention was designed for mid-level health workers (nurses and clinical officers) and employed an integrated guideline adapted to Malawian protocols for adults with respiratory conditions, HIV/AIDS, tuberculosis, and other primary care conditions and trained mid-level health workers in the use of the guideline through peer-led on-site training sessions [19]. During the period of the present study, LHWs were permitted to sit in on PALM PLUS training sessions at some sites.

### Data collection and outcome measures

Outcome data were obtained from routinely collected Ministry of Health records and included TB patients starting treatment in Zomba district on or after April 1, 2011 and whose treatment period ended on or before March 31, 2012. Cards were digitized and double entered by trained data entry clerks. Cases with no outcome recorded on the TB treatment card were traced back to the health center and updated using the outcome recorded in the TB registers. As a large number of cases also had no outcome noted in the TB register, TB treatment cards were reviewed and the final outcome updated based on the record of administered treatment.

TB outcomes were classified based on World Health Organization definitions [20]. The primary outcome of interest was the proportion of treatment successes, defined as the combined total of cases cured and completing treatment. Secondary outcomes of interest included: proportion of default cases, the proportions of success and defaults among patients co-infected with HIV, and weight change as a surrogate marker of clinical improvement.

### Sample size

An *a priori* sample size calculation was conducted to determine the number of patients needed per cluster, based on the binary outcome of TB treatment success, with an alpha of 0.05 and power of 0.80. We estimated a treatment effect size of a 0.15 increase in proportion in successful treatment over the 0.78 successful treatment rate for usual care based on published local treatment success rates and findings of studies with LHWs as adherence supporters in similar settings [21,22]. An intra-cluster correlation coefficient (ICC) of 0.1 was estimated as a mid-range value from a list of ICCs from similar studies in terms of intervention targets, outcomes, and units of randomization [23]. Given these parameters and a total number of 28 clusters available for randomization, we calculated a required sample size of 14 patients per cluster, for a total of 392 patients. Based on the number of TB notifications per year, a trial period of 1 year was anticipated to be sufficiently long to accrue this sample size.

### Statistical analysis

Analysis was by intention to treat, and results reported according to the consort guidelines for pragmatic and cluster randomized trials. Given the relatively small number of clusters (health centers), the effectiveness of randomization was evaluated through descriptive statistical comparisons of baseline patient characteristics. Inter-cluster correlations were calculated for outcomes of interest, with adjustment for unequal cluster sizes [24]. Univariate analysis of the primary outcome of interest, treatment success, and the preplanned subgroup analysis of treatment success by HIV status was conducted using chi square analysis of proportions adjusted for clustering [25] to reduce the risk of rejecting the null hypothesis in error, known to be elevated with generalized estimating equations (GEE) analysis with small sample sizes [26]. As the ICC for treatment success by HIV status was negative, an ICC of zero was assumed [27] and unadjusted chi square analysis was conducted. Given the inclusion of all three trial arms in the primary chi square analysis, odds ratios and confidence intervals were estimated using a GEE with trial arm as the only factor, in order to provide an approximate measure of effect size within the context of the overall chi square result. As odds ratios may be inaccurate with common events, odds ratios were converted to relative risks using the formula outlined in 1998 by Zhang and Yu [28].

Multivariate analysis of the primary outcome was conducted using GEE to account for clustering in assessing the effectiveness of the LHW intervention. The GEE utilized a binary logistic model with robust (sandwich) covariance estimator and an exchangeable correlation matrix to estimate the treatment effect as an odds ratio and to test for significance. Odd ratios were again converted to relative risk. The model for treatment success was built systematically. First, the independent effect of each pre-determined predictor on the outcome of interest was examined with only the predictor and trial arm in the model. Predictors with significant model effects were retained in the final model.

Four health centers accruing no patient level data were eliminated from analysis. This left one stratum with only one cluster, which precluded a stratified analysis. To adjust for any effects of the stratification variable, strata were assessed in the first step to be retained if significant. Pair-wise contrasts were conducted to assess the incremental effect of the LHW intervention over that of the PALM PLUS intervention alone. TB outcomes were not available for two cases in the control arm due to poor visibility of the TB card. These cases were excluded from the primary analysis and a sensitivity analysis conducted to assess their potential impact on the effect of the intervention.

Planned secondary analysis of proportion of default cases could not be undertaken due to the small number of events, <0.06 of cases. In addition, as both initial and final body weights were recorded in less than 5% of cases, analysis with weight change as a surrogate for clinical improvement could not be conducted. All analysis were two-tailed, with *p* values ≤ 0.05 considered significant.

## Results

### Intervention

In addition to the 7 TB focus LHWs trained as peer trainers who continued to provide patient care during the study period, a total of 49 general LHWs were initially reported by peer trainers to have completed the intervention training. This included all LHWs routinely providing TB care at PALM PLUS with LHW intervention sites at the start of the study and ranged from 3–12 per site, with all LHWs who started the training reported by peer trainers to have completed the training. However, this is likely an overestimate by 4–5, as 3 of 36 LHWs interviewed in a companion study reported that they had not completed the training. Pre-post knowledge assessment was not conducted due to concerns that LHW fears about ‘testing’ would negatively impact participation in the training. One trainer was laid off at the end of the first quarter, and seven trained LHWs lost over the course of the study (six transfers, one death). All LHW intervention sites reported meeting as a group, one to two times per quarter, beyond the initial training period to share experiences, discuss challenging cases, and to practice with the point-of-care tool at less busy centers.

### Baseline characteristics and study flow

Baseline characteristics were comparable across the three trial arms (see Table 2), with proportion of pulmonary TB cases relative to all TB cases, 0.58 in the control group, 0.74 in the PALM PLUS only group, and 0.80 in the LHW intervention group, the only significant difference (*p* = 0.027).

**Table 2:** **Baseline characteristics by trial arm**

**Factors**	**Control number (%)**	**PALM PLUS only number (%)**	**PALM PLUS and LHW intervention number (%)**	***p*** **value**
Cluster level				
Number of health centers	12	7	5	
ART initiation sites	3/12	2/7	1/5	
Cluster size mean (range)	6.7 (1–15)	9.7 (5–17)	6 (3–12)	
Patient level				
Number of patients	80	68	30	
Mean age in years (range)	37.0 (3–72)	39.3 (1–84)	38.7 (5–73)	.719
Women	40/80 (50)	38/68 (44)	17/30 (43)	.716
Incident TB cases	71/72 (99)	59/64 (92)	27/29 (93)	.398
Pulmonary TB cases	45/78 (58)	50/68 (74)	24/30 (80)	.027
HIV positive	29/63 (46)	24/56 (43)	11/22 (50)	.847
6-month weight recorded	3/80 (4)	2/68 (3)	1/30 (3)	.302

**Figure 1:**
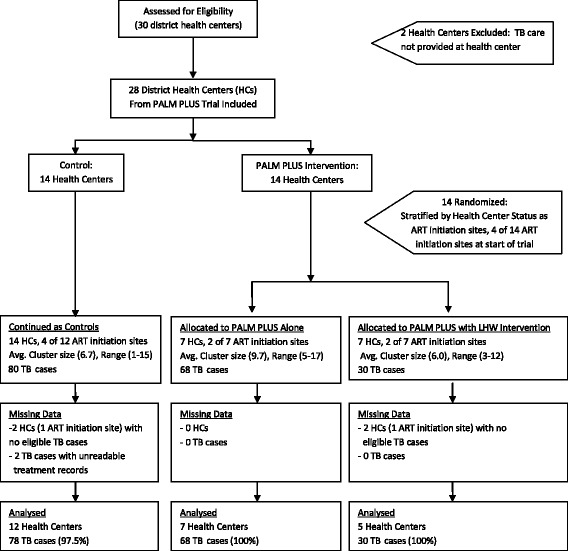
**Details of progress of clusters and individuals through trial.**

All 28 eligible health centers agreed to participate. Four health centers accrued no eligible patients and were eliminated from analysis (see Figure 1). TB outcomes by trial arm are shown in Table 3. Records were obtained for 178 eligible patients. Two cases could not be reconciled due to poor visual quality of the TB treatment cards (see Figure 1). Primary analysis was conducted with these two cases eliminated and a sensitivity analysis conducted to ascertain their potential impact.

**Table 3:** **TB treatment outcomes by trial arm**

**TB outcome**	**Control group number (%)**	**PALM PLUS only group number (%)**	**PALM PLUS and LHW intervention number (%)**
Cure	8/80 (10)	13/68 (19)	8/30 (27)
Treatment complete	38/80 (48)	31/68 (46)	13/30 (43)
[Success^a^]	[46/80 (58)]	[44/68 (65)]	[21/30 (70)]
Default	4/80 (05)	4/68 (06)	2/30 (07)
Lost to follow-up	15/80 (19)	11/68 (16)	3/30 (10)
Dead	11/80 (14)	9/68 (13)	4/30 (13)
Transfer out	2/80 (3)	0	0
Unknown	2/80 (3)	0	0

^a^Success defined as total of cases with outcomes of cure or treatment completion.

### Primary outcome

Results of a univariate analysis of the primary outcome are presented in Table 4. The overall proportion of treatment successes was 46/80 (0.58) in the control group, 44/68 (0.65) in the PALM PLUS alone group, and 21/30 (0.70) in the PALM PLUS with LHW intervention group. There was no evidence for effect of the intervention in the primary analysis adjusted for the effects of clustering (*p* = 0.578). Pair-wise contrasts conducted using GEE showed no significant difference between the three groups with relative risks and approximate 95% confidence intervals derived from GEE for the three comparisons as follows: PALM PLUS intervention alone relative to control RR 1.02, 95% CI (0.90–1.10), PALM PLUS with LHW intervention relative to control RR 1.04, 95% CI (0.96–1.11), and PALM PLUS intervention alone relative to PALM PLUS with LHW intervention RR 1.02, 95% CI (0. 95–1.08). Planned subgroup analysis of success among HIV status groups, also found no effect of the intervention (*p* = 0.91). Sensitivity analysis incorporating the two missing TB outcomes as successes or non-success had no effect on the findings.

**Table 4:** **Univariate analysis of primary outcome, treatment success, and subgroup analysis of treatment outcome by HIV status**

**Outcome**	**Control number (proportion)**	**PALM PLUS only number (proportion)**	**PALM PLUS and LHW intervention number (proportion)**	***p*** **value**	**ICC**
Proportion of success cases	46/80 (.58)	44/68 (.65)	21/30 (.70)	0.578	0.069
Success by HIV status					
HIV positive	19/46 (41)	16/44 (36)	9/21 (43)	0.911^a^	−0.045^b^
HIV negative	18/46 (39)	20/44 (45)	7/21 (33)		
HIV unknown	9/46 (20)	8/44 (18)	5/21 (24)		

^a^Adjusted for clustering.

^b^Negative ICC assumed to be zero therefore assessed with unadjusted chi square.

Consistent with the univariate analysis, analysis with GEE controlling for gender, TB type, and a TB type by intervention arm interaction term showed no significant effect of the intervention on treatment success (*p* = 0.76); see Table 5. Given the significant model effect found for the interaction between trial arm and TB type despite an overall non-significant result, we further investigated the interaction through a *post hoc* analysis using GEE stratified by trial arm. Results of the *post hoc* analysis agreed with those of the full GEE with a significant effect of TB type again found in the control arm only, RR for success in the control arm for pulmonary TB relative to non-pulmonary TB, 1.18, 95% CI (1.06–1.32). Again, sensitivity analysis incorporating the two missing TB outcomes as successes or non-success had no effect on the model or pair-wise contrasts.

**Table 5:** **Final model of primary outcome, treatment success**

**Model effects**	**Parameter estimate**	**Wald 95% CI**	***p*** **value**
Intercept	0.363	−0.400–1.126	0.352
Trial arm			
PALM PLUS and			
LHW intervention	−0.900	−1.920–0.120	0.084
PALM PLUS alone	−0.468	−1.486–0.551	0.368
TB type			
Non-pulmonary TB	−1.065	−1.815 to −0.314	0.005
Trial arm × TB type			
PALM PLUS and LHW intervention × non-pulmonary	1.312	0.341–2.283	0.008
PALM PLUS alone × non-pulmonary	0.435	−0.850–1.719	0.507
Gender			
Female	−0.636	−1.006 to −0.267	0.001

## Discussion

Although treatment success rates were higher in both intervention arms than in the control and highest in the PALM PLUS and LHW intervention group, the differences did not reach statistical significance. While this may represent a true finding, a number of other factors may be responsible for our failure to detect a significant effect of the intervention.

First, the lack of a significant difference may be an artefact as our projected sample size was not attained due to the loss of four health centers and decreased TB case notification rate during the trial period. The resultant loss of statistical power may have reduced our ability to detect an effect of the intervention. Second, it is possible that the trial’s pragmatic design contributed to the lack of effectiveness where a more intensively supported program may have been more successful. However, a heavily supported program would not be feasible in the resource-constrained Malawi health care system. Third, it is possible that a significant effect was not found due to a failure of implementation rather than lack of effectiveness of the intervention itself. In keeping with our choice of a pragmatic design, we choose not to include a formal implementation outcome evaluation in part due to the additional logistical burden it would impose, which is unlikely to be feasible under real-world implementation conditions (national scale up), but also due to concerns from LHWs around some forms of evaluation, particularly knowledge assessment. Elements of implementation were informally assessed where possible through feedback from participants during quarterly meetings with peer trainers, field visits conducted by the study team, and in a companion qualitative study (reported elsewhere) conducted to assess LHWs experience with the intervention and to identify areas for improvement before wider implementation. While together, these sources provide some information on implementation outcomes they do not provide enough information to ensure that a failure of implementation is not responsible for the non-significant findings. Although a formal implementation evaluation was not considered feasible for the present study as outlined above, a more thorough implementation evaluation could be possible with appropriate funding and tailoring of the implementation evaluation to address the logistical challenges and socio-cultural context of the implementation setting. Approaches that may facilitate a more thorough implementation evaluation in the settings such as the present study include: incorporating evaluation tasks into routine supervisory field visits and routine documentation where possible and educating participants about the purpose and of value evaluating implementation.

Several additional occurrences during the course of the trial may have diluted the impact of the intervention and contributed to the findings. First, loss of trained LHWs resulted in at least some provision of TB care by untrained workers which may have diluted the potential effects of the intervention. Second, treatment for TB/HIV co-infected patients changed part way through the trial reducing the pill burden on this group of patients which is a known risk factor for non-adherence [29,30]. Third, another potential reason for the lack of effect is that intervention failed to adequately address LHW needs or failed to address other factors important to adherence, such as patient-level factors, with the factors addressed insufficient to make a significant change in adherence. Finally, as the PALM PLUS intervention was rolled out to the rest of the district in the second half of the trial year and completed in most health centers in the fourth quarter of the present trial, it is possible that the impact of the PALM PLUS intervention on control arms may have contributed to the findings. However, as PALM PLUS was to be implemented throughout the district, our primary interest was in the additive effect of the LHW intervention as compared to the PALM PLUS intervention alone, with this comparison unaffected by the implementation of PALM PLUS during the present trial.

Consistent with other reports, we found significant model effects of gender with females more likely to successfully complete treatment [31]. In addition, we found a trend for an effect of TB type, significant only in the control arm, with lower success rates among non-pulmonary TB cases. A potential explanation for this finding based on our perception that non-pulmonary TB was generally less well understood by patients is that the enhanced patient education provided by intervention sites eliminated the effect by improving patient TB disease knowledge known to improve adherence [32,33]. Based on theoretical grounds and the findings of others [33], we hypothesized a significant effect of HIV co-infection. Interestingly, no significant effect of HIV status alone or as an interaction term was found. A variety of factors may have contributed to this finding including the relative low HIV positive rate for this region in the study population and the change to a combined TB/ART medication which equalized the pill burdens among the groups. The hypothesized role of TB type in adherence and the present finding suggest that further study is needed.

This study had several strengths. Randomization by health center rather than at the level of the individual provider minimized contamination. Inclusion of all district health centers increased generalizability of the results. Employing a pragmatic design revealed a number of challenges that suggest a need for increased supervision and support of trainers should this method of training be adopted.

There are several limitations to consider with our trial. Peer trainers may have been motivated in part by a desire to please the principle investigator who conducted their training and may have been regarded as a mentor. While this would not affect the outcomes of interest directly, peer trainers may have worked harder than might be otherwise expected. In addition, not all LHWs completed the training and several trained LHWs were lost during the course of the study, which may have diluted the effects of the LHW intervention. Finally, due to the nature of the ministry of health TB records, outcome assessors could not be blinded to health center. As a standardized approach was utilized in determining final outcomes, we feel that this is unlikely to have impacted the classification or findings.

## Conclusions

In our study, we did not identify a significant effect of our LHW KT intervention on TB treatment success; however, a trend for effectiveness was evident, particularly when combined with the PALM PLUS intervention. Given the urgent need for low-cost approaches to LHW training to ensure quality of services and improve health outcomes, further evaluation of tailored KT strategies as a means of LHW training in Malawi and other LMICs is warranted.
